# An Improved Near-Field Computer Vision for Jet Trajectory Falling Position Prediction of Intelligent Fire Robot

**DOI:** 10.3390/s20247029

**Published:** 2020-12-08

**Authors:** Jinsong Zhu, Lu Pan, Ge Zhao

**Affiliations:** 1 School of Mechatronic Engineering, China University of Mining and Technology, Xuzhou 221116, China; panmmeecumt@163.com (L.P.); zero166cmee@cumt.edu.cn (G.Z.); 2 Xu-gong Construction Machinery Group (XCMG) Research Institute, Xuzhou 221116, China

**Keywords:** intelligent fire robot, jet trajectory, near-field computer vision

## Abstract

An improved Near-Field Computer Vision (NFCV) system for intelligent fire robot was proposed that was based on our previous works in this paper, whose aims are to realize falling position prediction of jet trajectory in fire extinguishing. Firstly, previous studies respecting the NFCV system were briefly reviewed and several issues during application testing were analyzed and summarized. The improved work mainly focuses on the segmentation and discrimination of jet trajectory adapted to complex lighting environment and interference scenes. It mainly includes parameters adjustment on the variance threshold and background update rate of the mixed Gaussian background method, jet trajectory discrimination based on length and area proportion parameters, parameterization, and feature extraction of jet trajectory based on superimposed radial centroid method. When compared with previous works, the proposed method reduces the average error of prediction results from 1.36 m to 0.1 m, and the error variance from 1.58 m to 0.13 m. The experimental results suggest that every part plays an important role in improving the functionality and reliability of the NFCV system, especially the background subtraction and radial centroid methods. In general, the improved NFCV system for jet trajectory falling position prediction has great potential for intelligent fire extinguishing by fire-fighting robots.

## 1. Introduction

Public safety has always been an area of great importance for every country, and fire-fighting plays a significant role. In recent years, various types of fire extinguishing equipment have been developed towards intelligence and automation. Additionally, as the main equipment for fire-fighting, unmanned firefighting robots have attracted increasingly extensive attention [1,2]. Meanwhile, various types of fire-fighting robots have been designed and manufactured [3,4,5,6]. Among them, conventional fire-fighting equipment, such as crawler fire-fighting robots, have been widely adopted by the fire brigade. Table 1 lists the functional characteristics of several current mainstream fire robots in the world [7,8,9,10]. It is not difficult to find that the main role of current fire-fighting robots is to replace firefighters in order to enter some special environments to carry out fire-fighting work, such as high temperature, dense smoke, or narrow areas. The developed robots have made a very significant contribution to reducing the work intensity and casualties of firefighters. Furthermore, various vision sensors that are mounted on the robot have provided essential decision-making information for firefighters’ operations. However, the issue of final decision-making instruction still depends on the firefighter’s judgement of fire state, and the returned information from the visual sensor could only be viewed by firefighters. Under such a condition, the firefighting results would mainly depend on the firefighter’s execution ability and work experience. Factually, fire field conditions are changing constantly, for example, there would be combustion status [11], water supply pressure fluctuations [12], air heat convection, etc., which require the firefighters to continuously modify operations on firefighting robots. On the one hand, subtle position changes of the robot are difficult to be found by firefighters accurately during operation, which may directly cause inaccurate water jet trajectory. On the other hand, uninterrupted operation reduces fire extinguishing efficiency and leads to an increase in water consumption.

In the early 21st century, a two-nozzle with water and foam discharged at the speed of 5000 and 3000 L/min. respectively was developed by the Tokyo fire department and applied in firefighting [13]. Fang [14] designed a fire robot for railway tunnel fire that was capable of fire detection, alarm and extinguishment at the early stage. Kuo et al. [15] designed a fire-fighting robot with three flame sensors for smart building fire protection. Similarly, a fire extinguishing robot that is mainly used indoors, such as residences, offices, and high-rise buildings, was developed by Khoo [16]. The fire extinguishing robot was able to sense the flame and move to the fire location when the fire occurs in the house.

Furthermore, Fan et al. [17] proposed a method combining Gmapping SLAM and fire source recognition image processing algorithm, which realized the functions of autonomous navigation and fire source recognition and detection for fire-fighting robots. Researchers at Virginia Tech University have carried out a series of studies on fire-fighting robots. The fusion system of infrared stereo vision and LiDAR was used for fire recognition research under different visibility [18,19]. Meanwhile, the infrared stereo was also used for water jet trajectory identification and positioning research. Subsequently, the automatic control of fire monitor yaw and pitch angles was realized, and key technologies include the jet trajectory model based on experimental data and fire location algorithm of the fusion system.

A brief summary of the function realization and research directions of the fire robot visual sensors is given here. Firstly, fire recognition and positioning. The benefit from the continuous advancement of computer vision technology, monocular vision [20], binocular vision, infrared recognition, LiDAR, and unmanned aerial vehicles are widely used in fire recognition and location researches in various environments [21]. Subsequently, precise and continuous fire suppression. Related researches mainly focus on the establishment and modification of jet trajectory models [22,23]. However, position parameters of jet trajectory are subject to some random disturbances, such as the robot’s roll, pressure fluctuations in the water supply line, and random wind. Actually, the fire field environment is constantly changing. In order to adapt intelligent fire-fighting robots to various working environments, the robot vision system is developing in the direction of multiple sensors, fusion sensors, and sub-categories. The water jet trajectory’s continuous and accurate delivery of fire position is also gaining increasing attention.

The main contribution of this paper is to propose an improved NFCV method for jet trajectory identification and parameterization. Firstly, an improved mixed Gaussian background subtraction method was applied in jet trajectory identification based on the analysis of fire robot working environment. Subsequently, jet trajectory geometric features, including length and area ratio, were proposed in order to eliminate the false detection results. Furthermore, superimposed radial centroid method were developed to jet trajectory parameterization and feature extraction for falling position prediction. Finally, a comparative analysis between the given jet trajectory recognition and falling position prediction results and the results obtained through previous method was carried out.

The chapters of this paper are arranged, as follows: Section 2 briefly reviews previous work of the NFCV system, and several issues during application testing will be analyzed and summarized. Section 3 describes the improvement of the NFCV system, including: parameters adjustment of mixed Gaussian background method, jet trajectory discrimination, parameterization, and feature extraction of jet trajectory. Section 4 gives an introduction to the experiment and an analysis of some experimental results. Finally, some conclusions are given.

## 2. Previous Review

### 2.1. Near-Field Computer Vision

**Figure 1:**
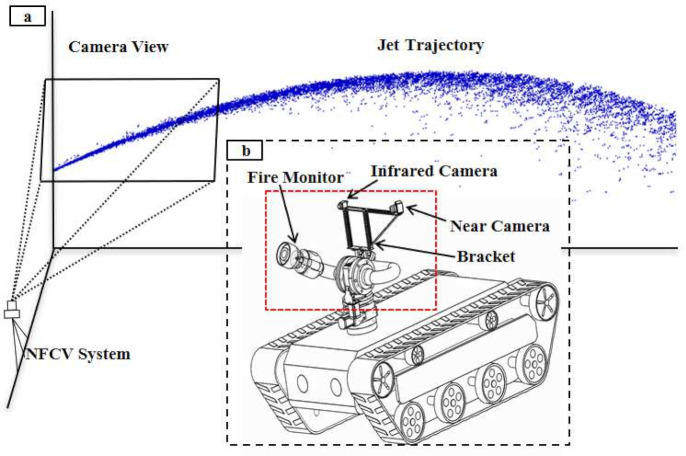
The structure of the near-field computer vision (NFCV) and fire robot. (**a**) NFCV; (**b**) fire robot equipped with fire monitor, red frame represents the visual system.

Regarding the research on the jet trajectory falling position prediction of fire monitor, the Near-Field Computer Vision (NFCV) method was proposed in our previous works [24]. It is mainly based on the following considerations: firstly, it is difficult to capture a long jet trajectory image completely, while capturing the initial image is relatively easy. Fortunately, the features that are contained in initial jet trajectory can also be used to predict the falling position. Therefore, the Near-field computer vision method was proposed, including hardware and software systems, as shown in Figure 1. Figure 1a,b present the structural diagram of the NFCV system and fire robot equipped with the system, respectively. In particular, an infrared camera is used in intelligent fire monitoring to detect fire target and so as to adjust range of yaw angle. Therefore, it is beyond the scope of this article.

The near-field camera captures jet trajectory image and sends it back to the computer during jetting, which includes image preprocessing, trajectory parameterization, and falling position prediction program developed based on C++ and OpenCV, as shown in Figure 1b. The main core functions include the following:

(1) Image preprocessing mainly includes perspective transformation, image enhancement, and trajectory segmentation. The near-field camera installed on the left side of fire monitor captures trajectory image from an oblique perspective, so perspective transformation is used in order to restore the image to the front view. Image enhancement is used to eliminate noise and highlight jet trajectory in the image. Jet trajectory is commonly white or bright, due to the fact that water tends to reflect light more easily. In the enhanced image, the brightness difference between jet trajectory and background are utilized in order to realize the jet trajectory segmentation.

(2) Trajectory parameterization mainly includes trajectory center position calculation and feature extraction. In the segmented image, trajectory parameterization is the prerequisite for feature extraction. However, the extracted jet trajectory from an image may still miss position parameters, due to residual noise. The mean position method was proposed and used to extract the jet trajectory position in the image and draw the trajectory curve. Suppose that the size of the image is m∗n, and g(i,j) represents the pixel value at (i,j) in the image; the jet trajectory ordinates in the binary image are expressed as:

(1) $$Y\left(i\right)=\frac{1}{a}\sum_{j=1}^{n}(n-j)\;g\left(i,j\right)=1$$

where *a* is the number of pixels that satisfies $g\left(i,j\right)=1$. Y(i) is the jet trajectory ordinate in each column of the binary image. Based on the acquired trajectory position data, the least square method was used for parametric fitting of trajectory. Furthermore, the jet trajectory feature, including the start-point slope (SPS) and end-point slope (EPS), were extracted, which are expressed as:

(2) $$SPS=\frac{dy}{dx}\left(x_{1}\right)$$

(3) $$EPS=\frac{dy}{dx}\left(x_{n}\right)$$

where *y* is fitted trajectory equation based on the jet trajectory position parameter in the image, $x_{1}$ and $x_{n}$ are the starting and ending point abscissa of the jet trajectory curve equation, respectively.

### 2.2. NFCV System Defects

In previous work, the proposed NFCV system has achieved good verification results in a static environment. However, many issues were discovered when the system was mounted on the fire-fighting robot during the application test, which are mainly manifested in:

Background interference: for walks of fire robots during the fire-fighting process, the background change of trajectory image captured by near-field camera is more dramatic. In particular, bright areas in the background may cause errors in trajectory segmentation. For example, bright sky background, standing water on the ground, etc.

Trajectory discriminant: meanwhile, there are other factors that cause deviations in jet trajectory detection. On the one hand, illumination changes cause sharp fluctuations in the brightness of jet trajectory in near-field image under outdoor environment. On the other hand, the state change of jet trajectory may lead to inconsistent brightness in image during jetting.

**Figure 2:**
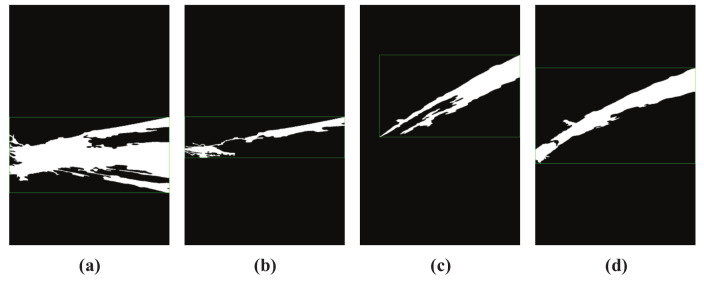
Different jet trajectory segmentation results. (**a**) wrongly trajectory result 1; (**b**) wrongly trajectory result 2; (**c**) wrongly trajectory result 3; (**d**) satisfactory trajectory result.

Figure 2 shows the segmented jet trajectory image. Figure 2a–c are the wrongly detected results of jet trajectory, and Figure 2d is the ideal jet trajectory segmentation result under the same camera parameters. It is not difficult to find that the false detection results mainly show that the background is mistaken for jet trajectory, such as Figure 2a, and part of the jet trajectory is removed as noise, such as Figure 2b,c.

**Figure 3:**
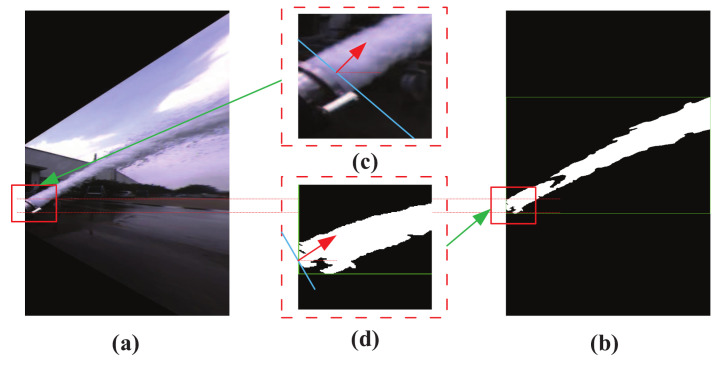
Disturbance in the process of jet trajectory feature extraction. (**a**) trajectory color image; (**b**) trajectory binary image; (**c**) fire monitor head color image, red arrow indicates the trajectory direction; (**d**) fire monitor head binary image, red arrow indicates the trajectory direction.

Feature extraction: generally, jet trajectory features extraction will be affected by the fire monitor head and the shape of trajectory. The color image of jet trajectory captured by the near-field camera is shown in Figure 3a. It is not difficult to find that the fire monitor head appears in the image and it was mistakenly detected as a part of jet trajectory, as shown in Figure 3b. Ideally, Figure 3c shows the theoretical incident angle of the jet trajectory, and it is indicated by the red arrow. In fact, in previous works, the leftmost side of the trajectory image is regarded as the starting position for calculating the jet trajectory incident angle, as shown by the red arrow in Figure 3d. Obviously, the mistakenly detected fire monitor head and application of the image leftmost position as the position of the incident angle may lead to inaccurate calculation results.

In general, when the proposed near-field vision system is mounted on a fire-fighting robot for functional testing, more complex light environment, changes of background state, and defective feature extraction methods may lead to the inaccurate prediction of the jet trajectory falling position. This phenomenon indicates that the application of the proposed NFCV system has defects in application on fire robots and it cannot meet the accuracy requirements of field testing and application. Therefore, improvement around trajectory misdetection, trajectory result discrimination, and trajectory feature extraction are the main focus of this paper.

## 3. Improved Works for NFCV

### 3.1. Improved Jet Trajectory Segmentation

Regarding test and application function realization of the proposed NFCV system on fire robot, the main improvements based on the foundation and analysis of previous work are, as follows.

#### 3.1.1. Background Subtraction for Jet Trajectory Detection

In the working process of fire robot, the background of the NFCV system is constantly changing. The segmentation of jet trajectory becomes difficult due to changes of bright areas or overall light environment. Therefore, in our works, the background subtraction method was used for trajectory segmentation on account of the continuous dynamic changes of jet trajectory during jetting. Furthermore, better jet trajectory identification parameters were set based on the actual working environment of the system. These mainly include: background variance threshold and update rate.

The gray value of any position of each frame that is captured by the NFCV system is constantly changing. In order to better adapt to the light environment and achieve accurate jet trajectory segmentation, the background of the near-field image is represented by multiple Gaussian background models. The gray value at pixel position (i,j) in the *t*th frame is represented as I${}_{t}$. I${}_{t}$ is constantly changing in each frame, and the gray value at each pixel is independent. The gray value change that is described above can be represented by a mixture model of *k* Gaussian distributions, and the probability distribution is expressed as:

(4) $$P\left(I_{t}\right)=\sum_{k=1}^{k}\omega_{k,t}N_{k}\left(I_{t},\mu_{k,t},C_{k,t}\right)$$

where, $N_{k}\left(I_{t},\mu_{k,t},C_{k,t}\right)$ represents the kth Gaussian distribution. $\mu_{k,t}$ and $C_{k,t}$ are the mean and variance, respectively, and $\omega_{k,t}$ is the weight of these distributions.

Furthermore, the probability function of Gaussian distribution can be expressed as:

(5) $$N\left(I_{t}\right)=\frac{1}{\sqrt{2\pi }\sqrt{C}}e^{-\frac{{\left(I_{t}-\mu \right)}^{2}}{2C}}$$

where, μ and *C*, respectively, represent the gray mean and variance of the preprocessed image. Furthermore, the weight update method of the mixed Gaussian background is expressed as:

(6) $$\omega_{k,t}=\left(1-\alpha \right)\omega_{k,t-1}$$

where, α represents the update rate of the weight, which is custom implementation based on OpenCV. When α = 0, the background would not be updated, and it is updated each frame when α = 1.

In our work, the variance threshold of background update is rather high. Firstly, when considering environmental light changes, such as sudden darkening or brightening, the jet trajectory may be submerged in other bright areas. Subsequently, in the jetting process, for the reflection of water on the ground, it is necessary to reduce the variance threshold of foreground detection in order to eliminate more interference areas. Thirdly, when the spray angle of the fire monitor is high, part of the jet trajectory would coincide with the sky, which is also an important factor in choosing a larger variance threshold.

The background update rate is also adjusted to be smaller, mainly based on the following considerations. Firstly, camera shake may increase the irregular false detection of the foreground. Secondly, falling water droplets peeled from the jet trajectory may be mistakenly detected as moving objects. Thirdly, in the continuous jetting process, there would be misdetection of certain micro-movements, such as clouds in the sky, and other objects that easily reflect light.

In general, when considering the actual working conditions of the NFCV system in application, the variance threshold and update rate of the background are applied to better achieve jet trajectory segmentation in the near-field image. Based on the analysis of factors affecting the jet trajectory segmentation results and the principle of mixed Gaussian background subtraction method, the background update variance threshold and update rate are set to be 20 and 0.1 in our works, respectively.

#### 3.1.2. Adequate Trajectory Discriminant

As we know, the ideal segmentation result of jet trajectory is complete and parabolic-like in the near-field image. However, the jet trajectory extracted by the parameter-adjusted background subtraction method combined with actual working condition may still have some false detections. For instance, objects in the background that are highly integrated with jet trajectory or abnormally moving objects in the near-field image. All of the above factors may cause over detection or weak detection of jet trajectory. Therefore, it is very necessary to judge the credibility of the extracted jet trajectory.

**Figure 4:**
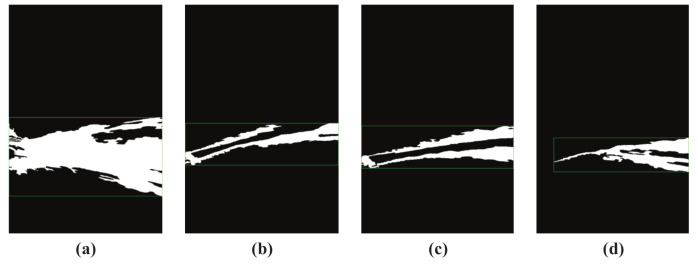
Misdetected results of jet trajectory. (**a**) over detection trajectory result; (**b**) weak detection trajectory result 1; (**c**) weak detection trajectory result 2; (**d**) weak detection trajectory result 3.

Figure 4 shows several misdetection results of jet trajectory. Figure 4a shows the over detection results of jet trajectory; it is not difficult to find that the area under jet trajectory is over detected. The reason for this is that the fire monitor cannot achieve long-distance spraying at the beginning of jetting due to insufficient kinetic energy of the jet trajectory. The sprayed water approximates a free fall in the NFCV system view field, and was detected as dynamic objects. Figure 4b–d are weak detection results of jet trajectory. Obviously, the jet trajectory center areas presented in Figure 4b,c have not been detected. The long-term stable jetting is the reason for this result. The center area of jet trajectory is absorbed as the background, because the pixels value in this area doesn’t change over time. Nearly half of the jet trajectory that is shown in Figure 4d was not detected, possibly due to drastic environmental light changes. Therefore, the above-mentioned jet trajectory detection results cannot be used in falling position prediction and they must be eliminated.

In our works, the jet trajectory length and area proportion features were proposed and used as the basis for validity judgment, and they are represented as:

(7) $$X_{rat}=\frac{R_{\max}-R_{\min}}{L}$$

(8) $$S_{\mathrm{rat}}=\frac{\sum_{i=0}^{S}I_{s}}{S}$$

where, *L* and *S* represent the width pixels and total pixels of the near-field image, respectively. $R_{\max}$ and $R_{\min}$ represent the start and end horizontal coordinates of jet trajectory in near-field image, respectively, and $I_{s}$ represents jet trajectory total pixels in near-field image.

In our works, for satisfactory jet trajectory discrimination, more than 800 images have been summarized in order to obtain accurate validity judgment threshold. For length proportion of jet trajectory, no less than 0.95 is regarded as a satisfactory judgment parameter. Similarly, the area proportion is set to be from 0.05 to 0.2. The range setting of above two parameters is mainly based on two considerations. On the one hand, jet trajectory that cannot be effectively extracted must be eliminated, as accurate falling position prediction is impossible. On the other hand, excessively strict judgment parameters may lead to continuous no jet trajectory detection results output and failure of the NFCV system, or even the entire intelligent fire monitor system cannot work effectively.

### 3.2. Trajectory Parameterization

In Section 2.2, issues regarding the trajectory parameterization and feature extraction methods of the proposed NFCV system are analyzed. Combined with previous work, secondary trajectory fitting methods are proposed based on the mean position method and radial centroid method. Firstly, the mean position method was used to construct the preliminary jet trajectory. Furthermore, based on the preliminary jet trajectory equation, secondary jet trajectory equation was established using the proposed radial centroid method, which can be used in more accurate description of jet trajectory state and feature extraction.

#### 3.2.1. Mean Position Method

As we know, pixel position of jet trajectory in the image is an extremely important prerequisite for trajectory parameterization. However, the boundary of obtained jet trajectory is usually not smooth, and it even has some holes. The light reflection of jet trajectory and water rapid flow are the main reasons for this phenomenon. Therefore, the mean position method was proposed in previous work for jet trajectory position acquisition and it has been briefly reviewed in Section 2.1.

**Figure 5:**
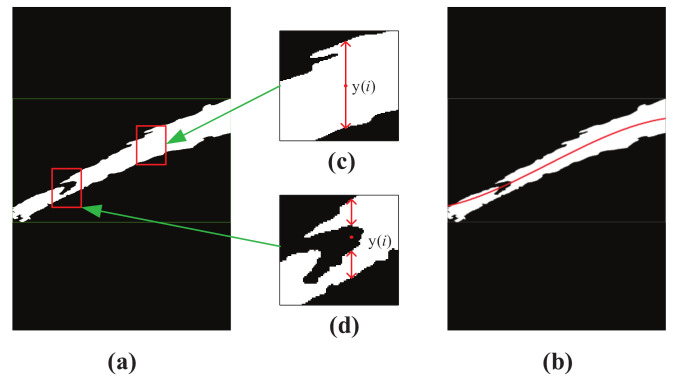
Example of mean position method results for binarized trajectory image. (**a**)binarized trajectory image; (**b**) trajectory curve; (**c**)trajectory center position 1, red double arrow indicates the trajectory in the binarized image; (**d**) trajectory center position 2, red double arrow indicates the trajectory in the binarized image.

Figure 5 shows the example of the mean position method results for binarized trajectory image. It can be seen that, for the non-smooth jet trajectory boundary, the center position could be accurately calculated, as shown in Figure 5. Fortunately, a satisfactory center position was obtained for jet trajectory cavity whose upper and lower jet trajectory positions are counted, even if partial jet trajectory is undetected. Figure 5 shows the calculation result of jet trajectory with holes.

Figure 5b shows the pixel coordinate position of jetting trajectory in the image, which is obtained through fitted jet trajectory results that are based on the proposed mean position method. Clearly, the fitted red curve cannot accurately express the true shape of jet trajectory due to the misdetection of fire monitor head, which corresponds to Figure 2a. Therefore, it is necessary to improve the trajectory parameterization method for more accurate trajectory parameter expression and feature extraction.

#### 3.2.2. Radial Centroid Method

A new innovative radial centroid method was proposed based on the trajectory data result of the mean position method for secondary jet trajectory fitting in order to more accurately obtain the jet trajectory pixels position in the image and improve the robustness of trajectory parameterization method caused by false detection.

Firstly, jet trajectory equation fitted by mean position method is expressed as:

(9) $$f\left(i,y(i)\right)=0$$

Subsequently, the normal of the jet trajectory equation is expressed as:

(10) $$G\left(x\right)=\frac{x-i}{f^{\prime }\left(i,y(i)\right)}+y\left(i\right)$$

Finally, center pixels position along normal direction of the jet trajectory is calculated as:

(11) $$\begin{array}{cc} Y\left(i\right)=\frac{1}{A}\sum_{j=1}^{n}\left(n-j\right) & G(i,j)=1 \end{array}$$

where, $f\left(i,y(i)\right)$ is the trajectory equation calculated by the least square method, G(i) is the normal equation of each point in the trajectory. *A* represents the number of jet trajectory pixels in the image along the normal direction and $Y\left(i\right)$ represents the ordinate average value of jet trajectory pixels in the image that is based on the proposed radial centroid method.

**Figure 6:**
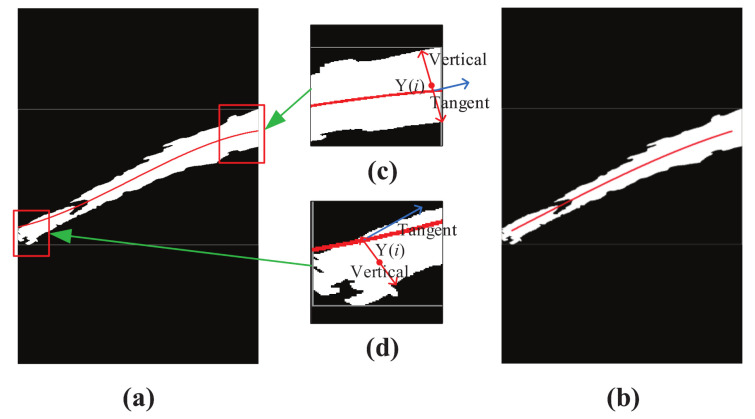
Example of radial centroid method results for binarized trajectory image. (**a**) binarized trajectory image; (**b**) trajectory curve; (**c**) calculation result of radial centroid method at the destination position of jet trajectory; (**d**) calculation result of radial centroid method at the origin position of jet trajectory.

Figure 6 shows the result of jet trajectory of a near-field image based on the radial centroid method, and the size of the near-field image is 600×900. When considering the computational timeliness of the NFCV system, the calculation step length of the radial centroid method is defined as 5 pixels. Furthermore, the calculation origin and destination of the radial centroid method are defined at 30 and 575 horizontal pixel position, respectively. Figure 6c,d show the calculation process schematic diagram of the proposed radial centroid method at the origin and destination of the jet trajectory, respectively. It can be seen that the new calculation position $Y\left(i\right)$ was not on the jet trajectory preliminary curve based on the mean position method. This shows that the center point pixel position of jet trajectory in the image has been corrected based on the new method. The secondary trajectory curve was drawn based on the updated position data, as shown in Figure 6b. Obviously, the new curve more accurately represents the original jet trajectory, and it is not sensitive to false detection objects, such as fire monitor head. Furthermore, the destination pixels position of the secondary trajectory curve does not exceed 575, which accurately ensures the EPS precision of the jet trajectory.

## 4. Experimental and Discussions

The improved near-field vision system was mounted on a fire robot and its performance in the factory environment is tested. Firstly, the experimental system was briefly explained, including the hardware structure and improved NFCV system workflow. Furthermore, experimental data and analysis results are given.

### 4.1. Experiment Setup

**Figure 7:**
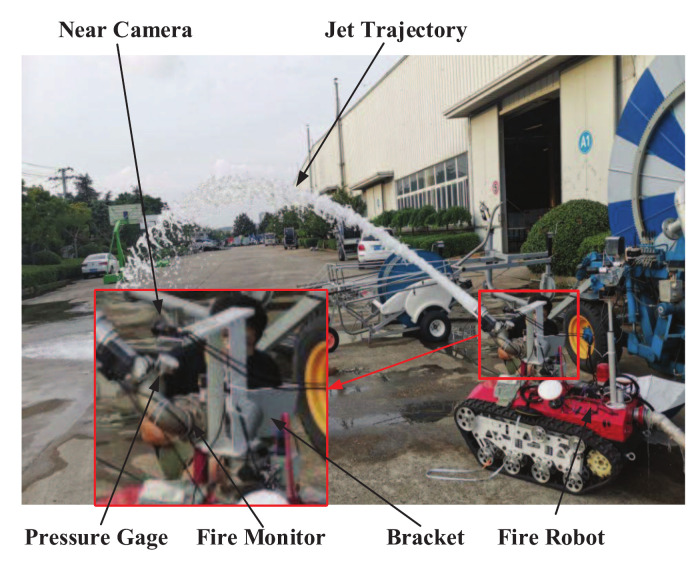
Structural diagram of the experimental platform.

The hardware system of the experiment mainly consists of the NFCV system and fire robot. Additionally, the experimental platform includes near-field camera, bracket, fire monitor, fire robot, and other auxiliary equipment, as shown in Figure 7. Furthermore, a near-field camera was connected to a personal computer with a self-designed image processing program, which is mounted 25 cm left of the fire monitor through a bracket. The selected fire monitor model is PS20–50, which is a common equipment in the market. The independently developed computer program could realize the yaw and pitch angle adjustment of the fire monitor through the development board. It must be pointed out that the NFCV system follows fire monitor through horizontal rotation. In other words, near-field camera is only used to capture the changes in the jet trajectory state that are caused by the pitch angle adjustment of the fire monitor.

**Figure 8:**
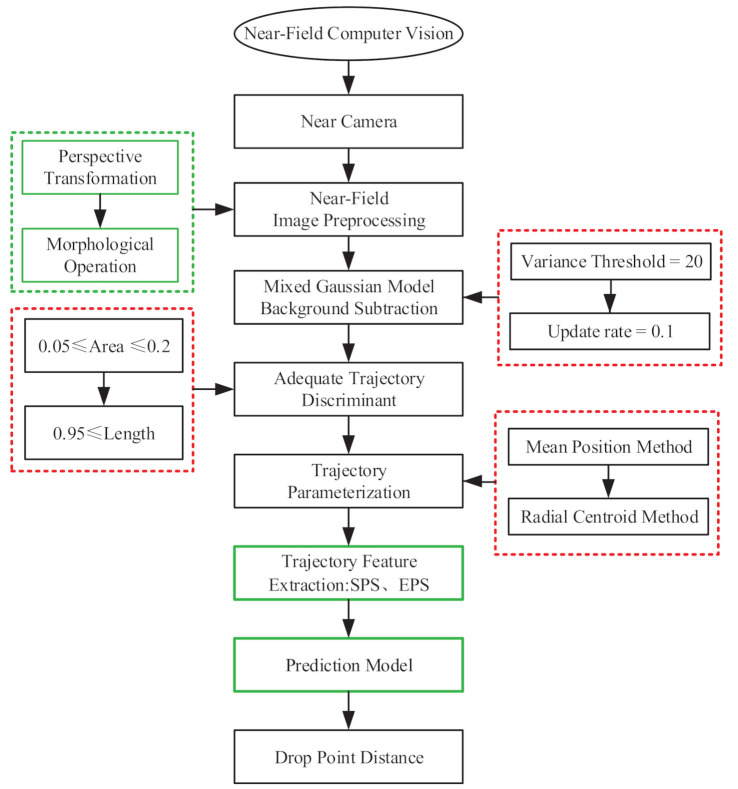
Workflow of the improved NFCV system, green and red frame represent previous and current work about fire robot, respectively.

The complete work process was expressed in Figure 8 in order to further illustrate the work process of the proposed near-field vision system and our latest work progress.

Image preprocessing: umage preprocessing is the first step of almost all vision systems, including perspective transformation, image morphology operations, and image enhancement operations. Particularly, perspective transformation is used to restore the front view of the jet trajectory. Other operations include camera distortion correction, perspective transformation matrix calibration, etc.

Jet trajectory segmentation: the segmentation method that is adapted to application test of the NFCV system is one of the focuses in this paper. Parameter optimization adjustment of the background subtraction method that is based on the mixed gaussian model is carried out in order to apply it to the collection environment of jet trajectory, which mainly involves variance threshold and background update rate; a detailed description can be found in Section 3.1.1.

Trajectory discrimination: unsatisfactory jet trajectory detection results may still occur due to other interference. Therefore, two important discriminant parameters, the length and area proportions, were proposed for ideal jet trajectory segmentation results; detailed information can be found in Section 3.1.2.

Trajectory parameterization: trajectory parameterization is an important guarantee for feature extraction, and existing issues are proposed and analyzed in Section 2.2. The radial centroid method was developed for trajectory parameterization and secondary jet trajectory drawing based on the mean position method, which is also one of our core works; a detailed description can be found in Section 3.2.

Prediction model: based on trajectory features and experimental data, the least square method was used in order to establish multiple regression model for falling position prediction of the jet trajectory. Additionally, the least squares method is commonly used for numerical regression, and thereby would not be repeated in this paper.

**Figure 9:**
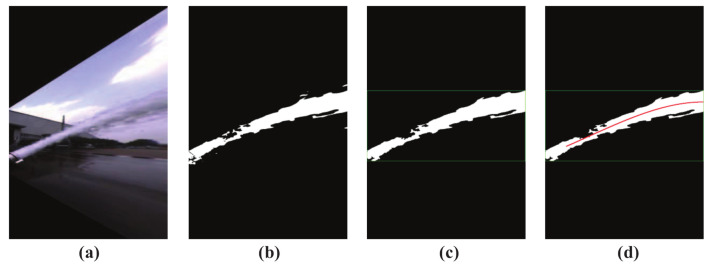
Processed image results after each step of the improved NFCV system. (**a**) preprocessed trajectory; (**b**) background subtraction; (**c**) trajectory discriminant; and, (**d**) trajectory parameterization.

Figure 9 shows the processed image results after each step of the improved NFCV system, and Table 2 lists the corresponding average processing time for each step. The improved background subtraction method and developed trajectory discrimination parameters are effective for the jet trajectory segmentation in the original image and a satisfactory fitting result of the jet trajectory equation was achieved based on the trajectory parameterization process with the radial centroid method.

### 4.2. Experiment Results and Analysis

**Figure 10:**
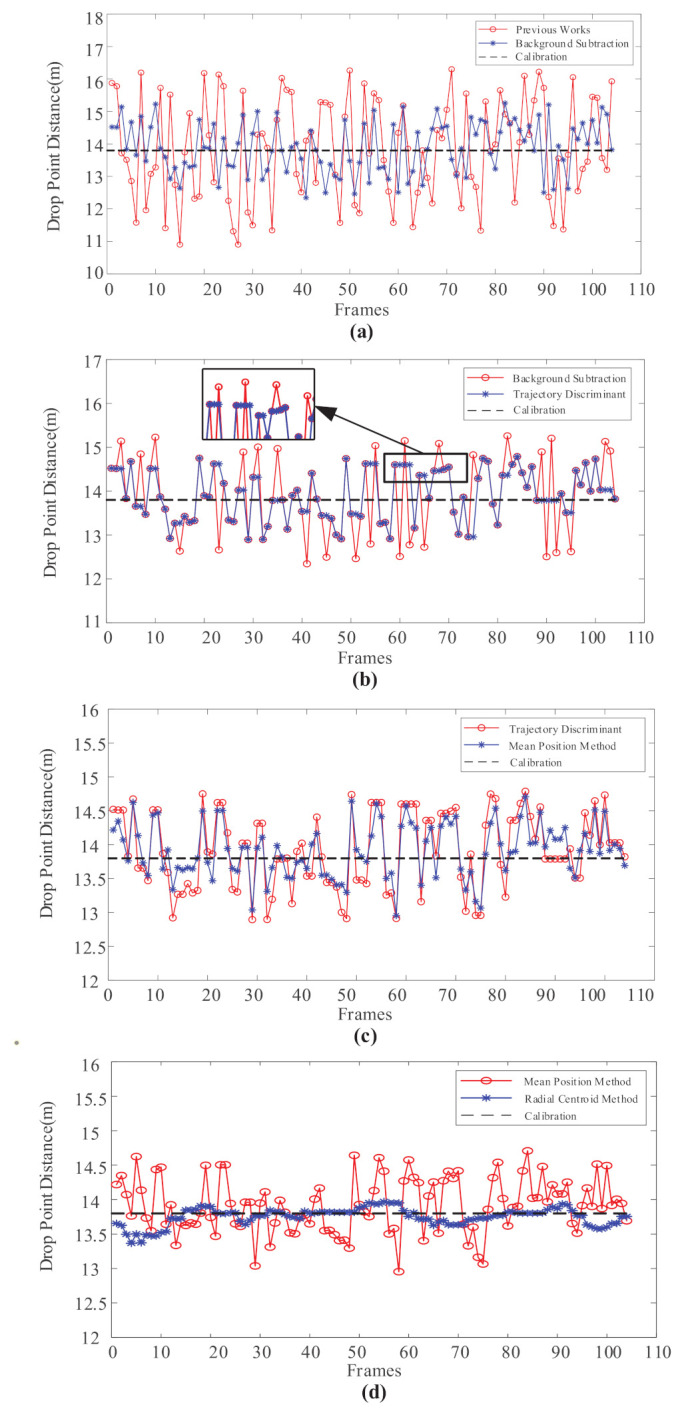
Comparison of jet trajectory falling position prediction results with Mixed Gaussian background subtraction, jet trajectory discrimination, mean position method, and radial centroid method were used incrementally. (**a**) Mixed Gaussian background subtraction; (**b**) jet trajectory discrimination; (**c**) mean position method; (**d**) radial centroid method.

The improved method for NFCV system includes Mixed Gaussian background subtraction, jet trajectory discrimination, and trajectory parameterization. In our works, a comparative analysis of the prediction results of falling points corresponding to the improvement work is carried out. The predicted results were compared with previous results in order to clearly show the improvement of results. In the experiment system, the water supply pressure of the fire robot was stably provided by a centrifugal pump. Furthermore, water outlet pressure at the fire monitor head was 0.115 MPa, since most of the pressure energy has been converted into kinetic energy in the internal pipeline of the fire robot. In general, the commonly used pitch angle of the fire monitor is 20–40${}^{\circ }$ during the fire extinguishing process. Furthermore, the abovementioned methods were used incrementally for falling position prediction with 104 captured jet trajectory images when the pitch angle of fire monitor was 35${}^{\circ }$, and the results are shown in Figure 10.

Obviously, the enhancement of every improvement work on prediction results is significant. Firstly, when compared with previous jet trajectory segmentation method, the mixed Gaussian background subtraction method of parameter optimization has significantly reduced the predicted results error from 1.36 m to 0.69 m; the comparison curve is shown in Figure 10a. Clearly, the mixed Gaussian background subtraction method is more suitable for jet trajectory segmentation of the NFCV system in the current testing environment. In particular, the higher variance threshold and slower background update rate improve the accuracy of the predicted results significantly. Figure 10b shows the comparison between the results obtained through the mixed Gaussian background subtraction method and after trajectory discrimination. It can be seen that the parameters of length and area proportion have effectively filtered relatively large incorrect prediction values. In the magnified box pointed by the black arrow, it is easy to find that the segmented jet trajectories that not satisfied the judgment parameter were eliminated by the developed method. The results manifested in the figure are the predicted results that have not been updated, as shown in the black frame. Among the 104 frames of captured images, approximately 21 frames were eliminated, accounting for 20.1%.

Figure 10c shows the comparison between results that were obtained by trajectory discrimination and mean position method increased. The mean position method was used for preliminary trajectory parameterization, which is simply used in previous work as well, as introduced in Section 4.1. Obviously, the mean position method slightly improves the prediction accuracy with average error reduced from 0.49 m to 0.34 m. Therefore, the radial centroid trajectory parameterization based on mean position method was necessary, and the comparison curve of predicted results is shown in Figure 10d. It can be clearly seen that the secondary parameterized trajectory after radial centroid method could realize accurate falling position prediction, with an average error of 0.10. Overall, the predicted position that is represented by the red dot in Figure 10a gradually approaches the black dotted line, which is the experimentally calibrated position of 13.8 m, with the improved method sequentially increased for the NFCV system.

**Figure 11:**
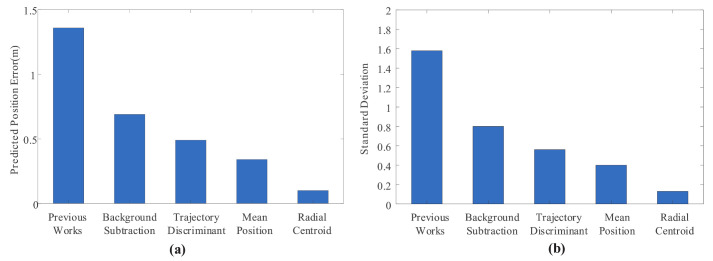
The mean and variance of prediction results for each improved method increased sequentially. (**a**) mean of prediction results; (**b**) variance of prediction results.

Figure 11 shows the mean and variance of prediction results for each improved method increased sequentially. Declining data results indicated that the improved method was positive and effective in improving the reliability of the NFCV system, especially the mixed Gaussian background subtraction method after parameter adjustment and the trajectory parameterization based on the radial centroid method. Furthermore, successively decreasing variance indicated that the stability of the NFCV system has also been further improved, which plays a positive role in improving and realizing the application feasibility of the system in intelligent fire robots.

**Figure 12:**
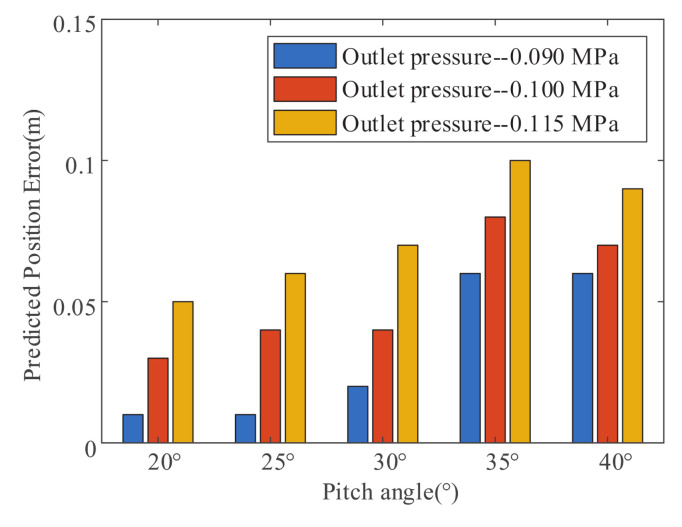
Falling position prediction error under different pitch angles and fluid supply pressures that are based on the improved NFCV system.

Figure 12 shows the jet trajectory falling position prediction error under different pitch angles and fluid supply pressures that are based on the improved NFCV system. It is not difficult to find that the falling position average error value increases with the supply pressure, and the maximum error is 0.09 m at 0.115 MPa of the fire monitor outlet pressure. Similarly, the pitch angle of the fire monitor is negatively related to average error, and the maximum error is around 35${}^{\circ }$. It needs to be pointed out that the error has little effect on the efficiency of fire extinguishing, because the fire extinguishing agent will spread out into an elliptical area with a large area at the terminal of the jet trajectory.

Table 3 provides the detailed results, mainly including the classification of prediction errors and comparison with previous methods. It was not difficult to find that the errors of the proposed improved methods were all less than 0.5 m, and more than half of the errors were less than 0.1 m. In other words, the improved NFCV system basically satisfies the performance requirement of intelligent fire robots for fire-fighting, and even for smaller fire spots. When compared with previous works, the improved method has played a huge role in improving the functionality and reliability of the NFCV system. Meanwhile, the improved algorithm has not increased image processing and program calculation time, which could fully satisfy no less than 1 Hz sampling frequency of the near-field camera.

## 5. Conclusions

An improved method for the NFCV system was proposed in this paper, including: the mixed Gaussian background method after parameter adjustment, jet trajectory discrimination, parameterization, and feature extraction of jet trajectory. The experimental results suggest that considering the complex light environment in the application of the NFCV system, higher variance threshold and slower background update rate play a positively significant role in reducing the false detection of jet trajectory. Furthermore, through the analysis of jet trajectory shape characteristics, the proposed jet trajectory discrimination method that is based on the length and area ratio parameters assisted the elimination of some stubborn false detection results. Finally, jet trajectory parameterization of radial centroid method was superimposed based on mean position method, which further increases the accuracy of the prediction results. The prediction error of no more than 0.5 m and more than 60% less than 0.1 m indicated that the improved NFCV system basically satisfies the performance requirement of intelligent fire robots for fire-fighting, and even for smaller fire spots.

Our future work mainly includes two aspects. On the one hand, it is necessary to further improve the prediction accuracy of the NFCV system in various practical environments. After all, there may be many interference factors, which have not been considered yet. On the other hand, the application of the NFCV system should be further expanded on an intelligent fire robot. For example, aiming and tracking of fire, combined with infrared vision, coping with the change of jet trajectory falling position due to the supply pressure fluctuation of fire robot, etc.

## Figures and Tables

**Table 1:** The functional characteristics of several current mainstream fire robots in the world.

Items	Model	Country	Sport Mode	Sensors				Highlight Function
				Infrared Vision	Vision Camera	LIDAR	UAV Vision	
1	ScrumForce	Japan	Wheel		✓		✓	Aerial and ground fused perspectives for fire detection
2	Colossus	French	Crawler	✓	✓			Firefighting for narrow terrain
3	WalkMan2.0	Italy	Walk	✓	✓	✓		Excellent fire environment awareness
4	THOR	America	Walk	✓	✓	✓		Smoke-filled firefighting
5	Thermite3.0	America	Crawler	✓	✓			Remote monitoring of dangerous fire scenes
6	RXRM50BD	China	Crawler	✓	✓			Fire detection and tracking

**Table 2:** The average processing time for each step of the improved NFCV system.

Items	Near-Field Image Preprocessing	Mixed Gaussian Model Background Subtraction	Adequate Trajectory Discriminant	Trajectory Parameterization	Average Processing Time
time (s)	0.24	0.12	0.16	0.41	0.93

**Table 3:** Analysis of prediction results of jet trajectory range.

Items	Error Range(m)	Amount	Average Magnitude of Error (m)	Mean Absolute Percentage Error (%)	Average Processing Time (s)
Improved	<0.1	64	0.04	0.30	0.93
	0.1–0.5	40	0.20	1.50	
	0.5–1.0	0	0	0	
	>1.0	0	0	0	
	Mean value	104	0.10	0.76	
Previous	<0.1	7	0.06	0.43	0.90
	0.1–0.5	13	0.32	1.50	
	0.5–1.0	18	0.73	2.32	
	>1.0	66	1.87	13.6	
	Mean value	104	1.36	9.86	
